# Full‐Mouth Digital Fixed Rehabilitation of a Deep‐Bite Patient Using a Minimally Increased Vertical Dimension: A Clinical Case Report

**DOI:** 10.1155/crid/2366489

**Published:** 2026-10-16

**Authors:** Mahdi Zahedi, Hakimeh Siadat

**Affiliations:** ^1^ Department of Prosthodontics School of Dentistry Tehran University of Medical Sciences Tehran Iran tums.ac.ir; ^2^ Department of Prosthodontics Dental Research Center Dentistry Research Institute School of Dentistry Tehran University of Medical Sciences Tehran Iran tums.ac.ir

## Abstract

This report presents the treatment of a 21‐year‐old patient suffering from severe Class II malocclusion, deep bite, anterior tooth fracture, and reduced vertical dimension of occlusion in a completely digital way. The treatment plan included cone beam computed tomography (CBCT), facial and intraoral scanning, virtual articulation, and computer‐aided design/computer‐aided manufacturing (CAD‐CAM). The provisional restorations were made to test the planned vertical dimension and occlusal relationship prior to fabrication of the final zirconia restorations. One year after completion of the treatment process, no signs of biological and mechanical failure, as well as occlusal stability and good results of esthetic and functional rehabilitation were found.

## 1. Introduction

Class II deep bite malocclusion is a multifactorial condition that can significantly compromise oral function and facial esthetics. Depending on the severity of the deformity and the patient′s needs, treatment may involve a combination of orthodontic, surgical, and restorative procedures to restore the vertical dimension (VD) of occlusion and provide predictable long‐term functional and esthetic results [1, 2].

Digital dentistry has revolutionized prosthodontics by enabling the precise and efficient fabrication of restorations. Beyond technical accuracy, digital planning enables clinicians to integrate soft‐tissue and facial landmarks, thereby facilitating treatment outcomes that are both functionally sound and esthetically harmonious [3, 4].

There are two principal modalities of three‐dimensional (3D) material fabrication in digital dentistry. Additive manufacturing is typically associated with 3D printing or sintering, whereas subtractive manufacturing is usually performed by milling [5].

CAD/CAM restorations are virtually designed based on digitized oral tissues. Digital reconstruction can be achieved either by a laboratory scanner that digitizes conventional casts or impressions, or direct intraoral impressions obtained with an intraoral scanner (IOS). Once the restoration is designed, the digital file is transferred to a computer‐aided manufacturing (CAM) unit [3, 6].

In conventional workflows, every step, from precise impression making and cast fabrication to the accurate transfer of occlusal relationships to a mechanical articulator, plays a critical role. Any deviation in this workflow can compromise the overall accuracy of the rehabilitation procedure. Inadequate communication between dentists, dental technicians, and patients can lead to uncertainty in prognosis and hinder the achievement of optimal esthetic results [7].

Facial scanners enable clinicians to create virtual patients, allowing them to assess dental treatments without requiring the patient′s physical presence. This technology facilitates the analysis of soft tissue and dental arch interactions in 3D space [8]. Additionally, cone beam computed tomography (CBCT) offers detailed volumetric imaging with high accuracy and low radiation exposure, making it the reasonable option for diagnosis and treatment planning. By merging CBCT files in Digital Imaging and Communications in Medicine (DICOM) format and digital impressions in Standard Tessellation Language (STL) files, volumetric and surface data are integrated, allowing precise diagnosis and prosthetic planning [5].

Contemporary digital workflows enhance predictability and overall treatment outcomes. The integration of facial scanning and IOS, mandibular motion analysis, virtual articulation, and CAD technology enables the creation of a virtual patient, supporting more efficient diagnosis, prosthetic planning, and treatment execution [9, 10].

The purpose of this clinical report is to describe the restorative management of a patient with a severe deep bite and Class II malocclusion. The treatment protocol consists of extraoral and intraoral examinations, diagnosis, digital treatment planning, diagnostic wax‐up, evaluation of alternative treatment options, full‐mouth rehabilitation procedures, and assessment of the maintenance phase.

### 1.1. Patient Information

A 21‐year‐old dentistry student with fractured anterior teeth and severe deep bite with 4‐mm overbite and 2‐mm overjet was referred to the prosthodontics department of Tehran University of Medical Sciences. His chief complaint is mainly esthetic difficulties and smile. The patient′s medical history was unremarkable, and he did not use any drugs or narcotics. In dental history, he had multiple amalgam fillings with recurrent caries, multiple discolored composite fillings, and fractured teeth. His maxillary lateral incisors were extracted due to an endodontic complication, and he had a splinted resin temporary bridge for Teeth Numbers 24, 25, 26, and 27 based on FDI two‐digit tooth numbering system (Figure 1).

**Figure 1:**
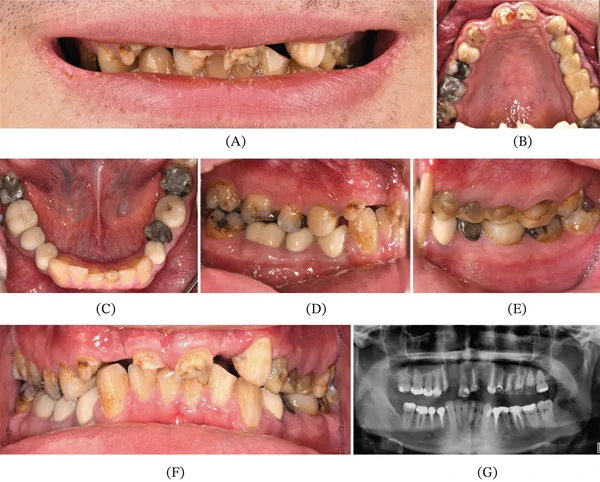
Pretreatment records of the patient. (A) Smile view. (B) Occlusal view of maxilla. (C) Occlusal view of mandible. (D) Frontal view. (E) Right lateral view. (F) Left lateral view. (G) Panoramic view.

### 1.2. Clinical Finding

Clinical examination revealed a Class II subdivision left molar and canine relationship associated with a severe deep bite and increased overjet without CR and MI discrepancy. A comprehensive periodontal evaluation demonstrated localized marginal gingivitis with no pathological mobility, and all remaining teeth were considered to have a favorable restorative prognosis. Examination of the temporomandibular joints and masticatory muscles revealed no pain, joint sounds, limitation of mandibular movement, or muscle tenderness. Phonetic evaluation and smile analysis demonstrated reduced incisal display and compromised facial esthetics associated with the decreased VD of occlusion. Limited restorative space was observed in the anterior region. Radiographic assessment, including panoramic and periapical views, confirmed adequate periodontal support and the absence of periapical or temporomandibular joint pathology. In clinical examinations and VD evaluation, it has been determined that the freeway space is 3.5 mm, and there is a chance of increasing VD to reach sufficient restorative space in the anterior segment [11]. The proposed increase in the VD of occlusion was determined through comprehensive evaluation of the patient′s physiologic rest position, freeway space, facial proportions, phonetics, and esthetics.

### 1.3. Timeline

Initial complaints were about esthetics and function of the tooth, and thus, after thorough clinical and radiographic examination (using CBCT), which was done within 1 month, an appropriate treatment strategy was determined. After that, the surgical and prosthodontic treatments were carried out in a staged approach for the following 3 months. Provisional rehabilitation was performed during the next 2 months, and finally, definitive prosthodontic rehabilitation took place in the next 4 months. The whole treatment process took around 12 months. Follow‐up examinations confirmed that there were no problems with prosthesis function and esthetics, and also, there were no biologic and mechanical complications.

### 1.4. Diagnostic Assessment

IOS (Medit i700 IOS; Medit Corp., Seoul, South Korea) of the upper and lower jaws was conducted using an anterior resin jig (Pattern Resin LS; GC Corp., Tokyo, Japan) to achieve a 1.5‐mm bite rise in centric relation. Centric relation was recorded using Lucia jig and verified by repeated recordings to ensure reproducibility. The selected VD was further validated during the provisional restoration phase over a period of 3 months, during which the patient adapted successfully without temporomandibular discomfort, muscle fatigue, speech difficulties, or functional limitations. The STL files of scan data were imported into design software (exocad DentalCAD software; exocad GmbH, Darmstadt, Germany), and a digital diagnostic wax‐up was completed based on the maxillary smile line. The completed wax‐up has been printed using a digital light processing (DLP) 3D printer (Anycubic Photon M3; Anycubic Technology Co. Ltd., Shenzhen, China) into a resin cast (Anycubic; Anycubic Technology Co. Ltd., Shenzhen, China) and a silicone index (Speedex putty; Coltene AG, Altstätten, Switzerland) taken from the wax‐up cast. The index was filled with temporary composite material (Techo Temporary; BISCO Inc., Schaumburg, Illinois, United States) and transferred to the patient′s mouth as a mock‐up. The mock‐up was evaluated for esthetics, phonetics, occlusal contact, and eccentric movement. The results were reviewed and accepted by the patient (Figure 2).

**Figure 2:**
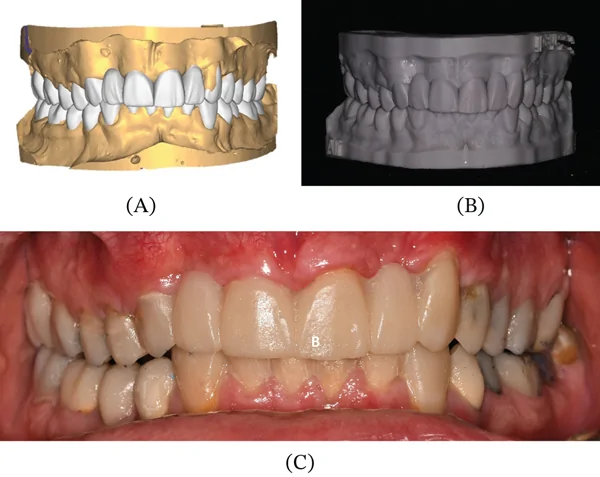
Diagnostic procedure. (A) Digital full wax‐up. (B) Printed waxed‐up resin casts. (C) Intraoral mock‐up views.

After taking a thorough history and radiographic examination, and regarding the patient′s chief complaints, the following problem list was obtained for the patient: severe deep overbite, discolored and fractured teeth with multiple caries and defective restoration, uneven incisal plane, loss of maxillary lateral incisors with insufficient bone width and attached gingiva, lower anterior segment overeruption, and past root canal treatment (RCT) insufficiencies.

Taking into consideration all the factors such as a fractured tooth, reduced vertical height, poor esthetics, and impaired function, it seemed that complete oral rehabilitation by means of full mouth prosthodontic therapy would be the best solution from a predictability point of view. This therapy offered restoration of occlusal stability, mastication function, facial support, esthetics, and vertical height while satisfying the requirements of the patient. Biological expenses of tooth preparation and possible future maintenance were discussed with the patient prior to treatment. Several alternatives were considered, such as orthodontic treatment, orthognathic surgery, orthodontic–prosthetic rehabilitation, and less radical forms of restorative treatment. Treatment limited to orthodontics would be ineffective in terms of restoring the severely damaged dentition and creating adequate conditions for restoration. Orthognathic surgery was also considered but rejected since the patient refused surgery. Orthodontic–prosthetic treatment was another possibility discussed with the patient, but he decided that the treatment should not be prolonged and include several stages. Ineffective would be conservative restorative procedures due to the extent of tooth wear, loss of VD, and the amount of restoration required.

The patient was unwilling to undergo orthodontic or orthognathic surgical treatment due to the long duration of therapy and consented only to prosthodontic reconstruction of his dentition. The treatment plan for the patient included full‐coverage all‐ceramic porcelain‐fused‐to‐zirconia (PFZ) restorations and laminate veneers (LV) for the mandibular anterior teeth, as well as implant‐supported crowns for the maxillary lateral incisors. Also, ceramic occlusal veneers (OV) in combination with deep margin elevation (DME) were considered for Teeth Numbers 15, 16, 37, and 35.

### 1.5. Therapeutic Intervention

Patient referred to the endodontics department for RCT of 11 and 31, and re‐RCT of 14 and 21. The patient was referred to the implant department for the placement of two Straumann NC implants (Institut Straumann AG, Basel, Switzerland) in Positions 12 and 22, along with autogenous and allograft bone reconstruction (Figure 3).

**Figure 3:**
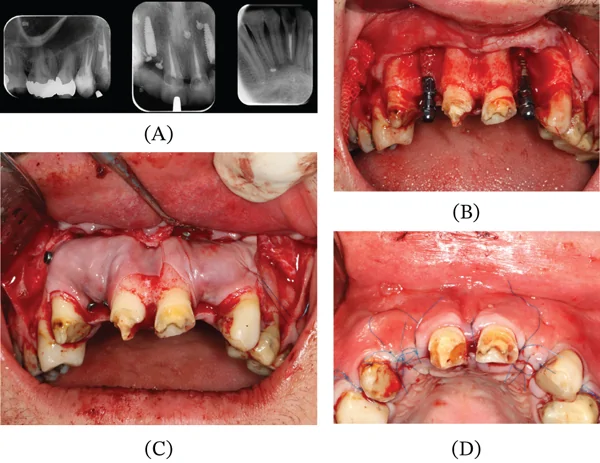
Preprosthetic treatments. (A) RCTs of Teeth Numbers 14, 11, 21, and 31. (B) Implant insertion surgery. (C) Allograft placement. (D) Flap closure using membrane.

After 5 months from the implant insertion, the restorative phase was initiated with the construction of indirect post patterns (Pattern Resin LS; GC Corp., Tokyo, Japan). The posts were cast to Ni‐Cr alloy (Damcast Ni‐Cr alloy; Damcast Medical Technology Co. Ltd., Beijing, China) and cemented with glass ionomer cement (GC Fuji I; GC Corp., Tokyo, Japan).

After cementation of Ni‐Cr casting posts, the DME technique [12], in conjunction with immediate dentin sealing (IDS) [13, 14] and BioBase [14, 15], was used to build the substructure for OV for Teeth Numbers 15, 16, 37, and 35. After isolation with a rubber dam and amalgam removal, the Tofel Maier matrix was used to box the teeth, and a layer of dentin bonding agent (All‐Bond Universal; BISCO Inc., Schaumburg, Illinois, United States) was added to the whole dentin area. Two sections of polyethylene fiber‐reinforced ribbon (Ribbond Inc., Seattle, Washington, United States) were placed on the pulpal floor and axial wall of the teeth to reduce composite shrinkage. Subsequently, a posterior bulk‐fill composite (Aelite LS Posterior; BISCO Inc., Schaumburg, Illinois, United States) was applied and cured in layers to shape the substructure. Finally, the composite layer was polished, and the rubber dam was removed (Figure 4).

**Figure 4:**
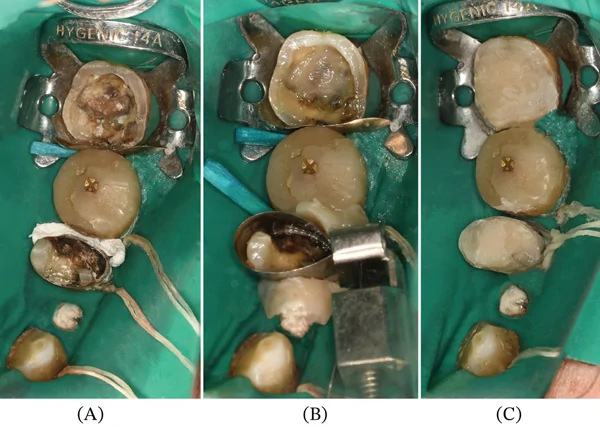
Deep margin elevation technique. (A) Isolation using a rubber dam. (B) Ribbond placement on the axial and pulpal floor. (C) Layer‐by‐layer composite filling.

Final preparation of full coverage restoration for maxillary and mandibular arch completed with deep chamfer finish lines [16] for better internal fitness. LV preparation with a butt joint [17] design was completed for mandibular anterior teeth. A three‐zero retraction cord (Ultrapak; Ultradent Products Inc., South Jordan, Utah, United States) was inserted into the sulcular area, and a layer of retraction paste (Astringent Retraction Paste; 3M Oral Care, St. Paul, Minnesota, United States) was added into the sulcus. After 5 min, the astringent paste was rinsed thoroughly, and all the teeth were dried. Implant‐level scan bodies (CARES NC Mono Scanbody; Straumann CARES, Institut Straumann AG, Basel, Switzerland) were firmly secured and torqued to 15 N. cm.

An IOS was taken with Medit i700 (Medit Corp., Seoul, South Korea), and a face scan was taken with Shining 3D face scanner (Shining 3D Tech Co. Ltd., Hangzhou, China). To record the bite in centric relation, after repeating the mock‐up process for posterior teeth, an anterior bite registration jig was formed with acrylic resin to record both the VD and CR simultaneously.

To record the patient′s facebow and determine the accurate condylar angle, a full‐face CBCT scan with a large field of view was conducted. Reasons behind the use of CBCT included the necessity of using 3D imaging for complete diagnostic analysis and implant stability evaluation. Selection of the field of view was done keeping the diagnostic needs into consideration and taking the as low as reasonably achievable (ALARA) principle into account. Compared with conventional computed tomography used in medicine, CBCT provides lower doses of radiation exposure and adequate imaging for dental purposes. The data collected from the IOS, facial scan, and CBCT were then superimposed automatically with best fit alignment using 3D face scan software from Shining 3D Technology Co. (Figure 5). After the superimposition, the combined data were exported from the face scan software in CAD file format and imported into the design software (exocad DentalCAD software; exocad GmbH, Darmstadt, Germany).

**Figure 5:**
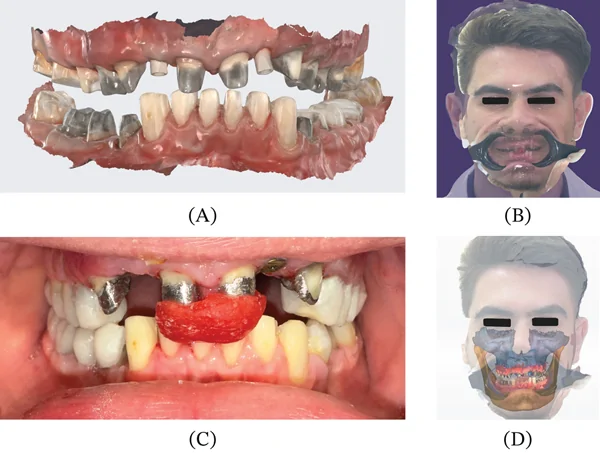
Final digital impression. (A) Intraoral scan of the prepared arch and implant scan body. (B) Face scanning with smile and retracted lips. (C) CR and VD determination using an anterior bite registration jig with a posterior teeth mock‐up. (D) Superimposition of data gathered from IOS, face scan, and CBCT.

In exocad, all datasets were combined and the virtual articulator (ARTEX CR; Amann Girrbach AG, Koblach, Austria) in Expert mode used for the virtual mounting of the patient′s scans. Firstly, the patient′s true condyle was aligned with the condyles of the virtual articulator. Next, the maxillary arch occlusal plane was transferred into exocad based on the facebow data collected from the CBCT scan (Figure 6A). Once the virtual maxillary cast was positioned on the virtual articulator, the condylar angles were adjusted. These adjustments were made using the values obtained from the patient′s CBCT scan, as well as those calculated with Hanau′s formula [18] within the virtual articulator settings. The correctness of superimposing the datasets was tested through matching the virtual contact point of the teeth to the clinical contact point and by ensuring that the superimposed maxillomandibular relation was reproducible. The various errors that could arise from registration such as patient movements during scanning and the errors caused by the scanner itself were eliminated using scanning procedures. This process ensured accurate alignment and representation of the patient′s dental anatomy in the virtual environment (Figure 6B).

**Figure 6:**
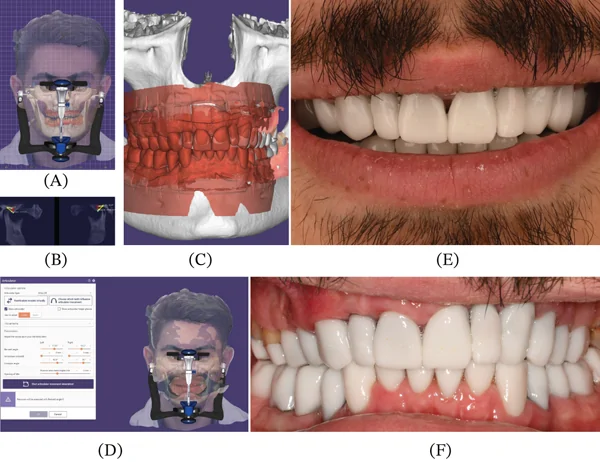
Virtual mounting and provisional design. (A) Virtual mounting of the maxillary arch using CBCT. (B) Adjustment of condylar and Bennet angle of virtual articulator using condylar angle from CBCT and Hanau′s formula. (C) Provisional digital design (skull view). (D) Provisional digital design (superimposed view). (E) Provisional try in smile view. (F) Provisional try in frontal view.

Provisional restorations were designed and positioned based on the patient′s facial scan data. After the provisional restoration design was approved, the restorations were 3D‐printed using a DLP printer (Anycubic Photon M3; Anycubic Technology Co., Shenzhen, China) and then transferred intraorally for evaluation. The provisional restorations were cemented for 2 months using Choice temporary resin cement (BISCO Inc., Schaumburg, Illinois, United States), and occlusal adjustments were made (Figure 6C–F). An IOS of the provisional restorations in occlusion was obtained and imported into exocad software for the design of the final restorations. The full‐coverage final restorations were then fabricated by replicating the design of the provisionals and applying porcelain veneers.

A cusp‐marginal ridge occlusal scheme was established, with stable bilateral posterior contacts in centric occlusion and anterior guidance providing posterior disclusion during excursive movements. Canine guidance was developed to eliminate posterior interferences during lateral excursions. Occlusal contacts were checked throughout the provisional stage, as well as the definitive one, using the virtual articulator and 40‐*μ*m articulating paper. In the provisional stage, the esthetic appearance, phonetics, occlusal stability, masticatory efficiency, temporomandibular joint comfort, and adaptation to an increased VD by the patient were all periodically checked.

Full‐coverage restoration frameworks as seen in Figure 7 were fabricated from zirconia (Audental Bio‐Material Co. Ltd., Shenzhen, China). After confirming the proper fit of the frameworks through radiographic evaluation, the final restorations were prepared by layering with IPS e.max Ceram (Ivoclar Vivadent AG, Schaan, Liechtenstein) in the shade B1. For the LV and OV cases, IPS e.max Press LT (Ivoclar Vivadent AG, Schaan, Liechtenstein) was used, followed by e.max Ceram layering. Once the ceramic layering was complete and an intraoral try‐in was conducted, the restorations were glazed and finalized for delivery.

**Figure 7:**
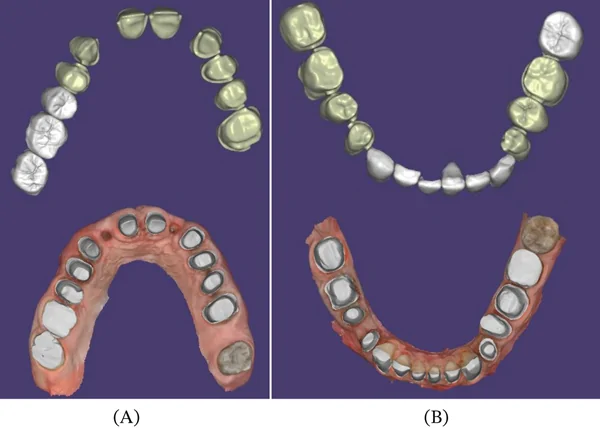
Frameworks design and margins detection. (A) Maxilla. (B) Mandible.

Full‐coverage restorations were cemented using Glass Ionomer cement (GC Fuji I; GC Corporation, Tokyo, Japan), laminate restorations were cemented with resin cement (Choice II resin cement; BISCO, Schaumburg, Illinois, United States), and OV were cemented using a self‐adhesive resin cement (Theracem; BISCO, Schaumburg, Illinois, United States) (Figure 8). Finally, an occlusal guard was fabricated and delivered to the patient to protect the ceramic restorations.

**Figure 8:**
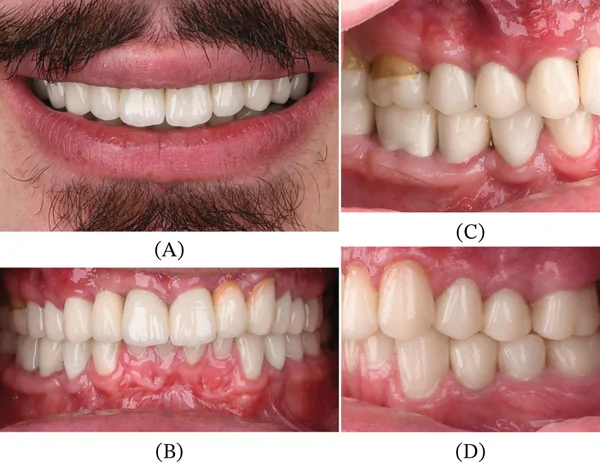
Delivery and cementation. (A) Smile view. (B) Frontal View. (C) Right lateral view. (D) Left lateral view.

### 1.6. Follow‐Up and Outcomes

The patient was reviewed at regular follow‐up appointments after delivery of the definitive restorations in 1, 3, 6, and 12 months. Clinical and radiographic (Figure 9) examination demonstrated stable occlusion, satisfactory marginal adaptation, no sign of peri‐implant disease and bone loss, and no biological or mechanical complications. The patient reported significant improvement in masticatory efficiency, speech, comfort, and smile esthetics and expressed a high level of satisfaction with the treatment outcome. At the latest follow‐up, the prostheses remained functional and stable, with no signs of restoration failure or occlusal instability.

**Figure 9:**
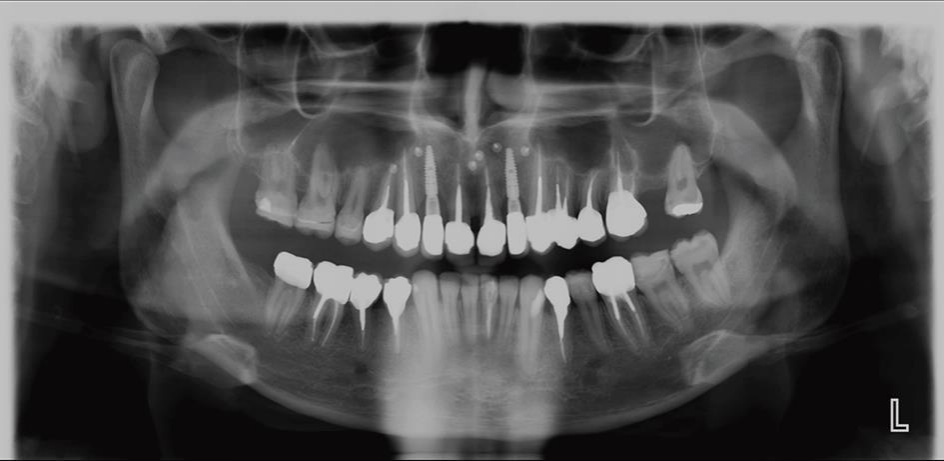
Follow‐up radiographs demonstrate satisfactory adaptation of the restorations with no radiographic signs of pathology.

## 2. Discussion

Severe deep bite combined with Class II malocclusion is a challenging condition, particularly in adults, where esthetic and functional demands are high. Traditionally, managing such cases often involves orthodontic or surgical interventions, which can be lengthy and demanding. In the present case, the patient preferred to avoid these extended treatments and opted for a prosthodontic‐only approach.

CAD‐CAM–assisted digital workflows have become an effective alternative to conventional prosthodontic procedures. Their advantages include greater clinical efficiency, enhanced patient comfort, reduced dependence on conventional stone casts, and improved treatment predictability. A notable feature is the integration of 3D meshes from various imaging modalities to create a virtual patient, enhancing treatment planning and communication. However, challenges include the costs of technology acquisition and a learning curve. Additionally, IOS demonstrates greater accuracy than conventional impressions, with studies showing CAD‐CAM crowns from IOS achieving marginal gap values under 60 *μ*m, whereas those from conventional impressions can exceed 183 *μ*m [19].

Evidence from a recent systematic review suggests that the conventional workflow continues to be regarded as the reference standard despite the growing adoption of digital techniques. Nonetheless, full digital workflow proves to be at least as effective as conventional and hybrid workflows in terms of time efficiency, production costs, precision, and patient satisfaction, especially for monolithic restorations [20].

The use of digital dentistry in this case significantly enhanced the treatment planning process. By integrating facial and IOSs with CBCT data, we created a comprehensive virtual model of the patient. A study by Yang et al. [21] found that CBCT is effective for analyzing two virtual dental patients. It concluded that a smartphone‐based facial scanner offers trueness like an industrial scanner, despite lower precision. The nose‐based integration technique was more accurate than the virtual facebow technique, which needs specialized equipment. Overall, compiling this digital data improved communication between clinicians and technicians, enhancing diagnostic procedures and treatment planning [22].

When all these tools have integrated, precise evaluation of soft tissue interactions, condylar angles, and occlusal relationships is possible. By using a virtual articulator and superimposing all relevant datasets, the team could plan both provisional and final restorations with confidence, reducing clinical uncertainty and ensuring predictable outcomes.

The restorations were created using both additive and subtractive manufacturing technologies [23]. Additive manufacturing was used to fabricate the resin‐based provisional restorations, whereas the definitive zirconia restorations were produced using CAD‐CAM milling to achieve precise fit and optimal marginal adaptation [19]. The completed rehabilitation resulted in stable occlusion, satisfactory esthetic integration, and a high level of patient satisfaction during the follow‐up period [24].

Porcelain layered zirconia was chosen as the final restorative material due to the proper combination of esthetic and mechanical qualities of that ceramic. Although the ceramic is known for its rather high elastic modulus (and hence increased transfer of the occlusal force to the supporting teeth and implant structures), it has outstanding flexural strength and fracture resistance; therefore, this ceramic would be the right choice for the patient with strong masticatory function and full‐mouth rehabilitation. In the case in question, severe deep bite of the patient and thus the expected strong load required a material that could resist strong occlusal forces and have sufficient mechanical endurance for a long time [7].

The present report presents the successful implementation of a completely digital protocol in one patient and proves its technical feasibility for management of a challenging prosthodontic rehabilitation procedure. Nonetheless, it is important to mention that the results should not be extrapolated widely since they are derived from a single patient and with a short follow‐up period. Although favorable short‐term results in both functional and esthetic terms have been achieved, it would be necessary to conduct additional studies with different patients to draw any more general conclusions.

## 3. Summary

This case demonstrates the value of a fully digital workflow for effectively managing complex rehabilitation processes. The integration of CBCT imaging, facial scanning and IOS, virtual articulation, and CAD/CAM technology facilitated accurate diagnosis, efficient treatment planning, and precise prosthesis fabrication. In challenging cases that demand both functional stability and esthetic excellence, digital workflows offer a reproducible, efficient, and highly predictable approach that may be difficult to achieve with conventional techniques.

## Author Contributions

**Mahdi Zahedi:** conceptualization, methodology, investigation, clinical management, data curation, writing – original draft, writing – review & editing, supervision. **Hakimeh Siadat:** conceptualization, methodology, investigation, clinical management, data curation, writing – original draft, writing – review & editing, supervision. Both authors contributed equally to this work.

## Funding

No funding was received for this manuscript.

## Disclosure

All authors have read and approved the final version of the manuscript. This case was done in prosthodontics department and dental implant department of Tehran University of Medical Sciences.

## Ethics Statement

Ethical approval was not required for this single anonymized case report according to institutional policy. Written informed consent for publication was obtained from the patient.

## Consent

Written informed consent was obtained from the patient for publication of this case report and the accompanying clinical images. The patient′s eyes were masked in all facial photographs to protect privacy.

## Conflicts of Interest

The authors declare no conflicts of interest.

## Supporting information


**Supporting Information** Additional supporting information can be found online in the Supporting Information section. 

## Data Availability

The data that support the findings of this study are available from the corresponding author upon reasonable request.

## References

[1] Ergun G. and Yucel A. S. , Full-Mouth Rehabilitation of a Patient With Severe Deep Bite: A Clinical Report, Journal of Prosthodontics. (2014) 23, no. 5, 406–411, 10.1111/jopr.12113, 24393501. 24393501

[2] Jeyaraj P. and Chakranarayan A. , Rationale, Relevance, and Efficacy of “Surgery First, Orthodontics Later” Approach in the Management of Cases of Severe Malocclusion With Skeletal Discrepancy, Annals of Maxillofacial Surgery. (2019) 9, no. 1, 57–71, 10.4103/ams.ams_272_18, 31293931. PMC6585223 31293931

[3] Garaicoa J. , Jurado C. A. , Afrashtehfar K. I. , Alhotan A. , and Fischer N. G. , Digital Full-Mouth Reconstruction Assisted by Facial and Intraoral Scanners: A Case Report and Technique Description, Applied Sciences. (2023) 13, no. 3, 10.3390/app13031917.

[4] Bessadet M. , Drancourt N. , and El Osta N. , Time Efficiency and Cost Analysis Between Digital and Conventional Workflows for the Fabrication of Fixed Dental Prostheses: A Systematic Review, Journal of Prosthetic Dentistry. (2025) 133, no. 1, 71–84, 10.1016/j.prosdent.2024.01.003, 38302291. 38302291

[5] Lanis A. , Gallucci G. , and Pedrinaci I. , Full Mouth Oral Rehabilitation of a Severely Worn Dentition Based on a Fully Digital Workflow, Journal of Esthetic and Restorative Dentistry. (2023) 35, no. 4, 596–608, 10.1111/jerd.13020, 36715027. 36715027

[6] Segundo Â. R. , Saraiva S. , de Castro C. , Sesma N. , Bohner L. , Andretti F. L. , and Coachman C. , CAD–CAM Natural Restorations—Reproducing Nature Using a Digital Workflow, Journal of Esthetic and Restorative Dentistry. (2023) 35, no. 7, 993–1000, 10.1111/jerd.13028, 36815432. 36815432

[7] Qiu A. , Xu L. , Zhang Y. , Chen K. , Fang S. , Zhang Y. , Zhong L. , and He R. , Digital-Assisted Multidisciplinary Treatment for Complex Occlusal Rehabilitation: An 18-Month Follow-Up Case Report, BMC Oral Health. (2024) 24, no. 1, 10.1186/s12903-024-04574-2, 39026272. PMC11264705 39026272

[8] Park J. H. , Lee G. H. , Moon D. N. , Yun K. D. , Kim J. C. , and Lee K. C. , Creation of Digital Virtual Patient by Integrating CBCT, Intraoral Scan, 3D Facial Scan: An Approach to Methodology for Integration Accuracy, Journal of Craniofacial Surgery. (2022) 33, no. 4, e396–e398, 10.1097/SCS.0000000000008275. 36041095

[9] Joda T. , Balmer M. , Jung R. E. , and Ioannidis A. , Clinical Use of Digital Applications for Diagnostic and Treatment Planning in Prosthodontics: A Scoping Review, Clinical Oral Implants Research. (2024) 35, no. 8, 782–792, 10.1111/clr.14230, 38140771. 38140771

[10] Abduo J. and Rasaie V. , Digital Workflows in Prosthodontics, Australian Dental Journal. (2025) 70, no. S1, S146–SS58, 10.1111/adj.70005. PMC12747636 40952009

[11] Mehta S. P. , Gupta N. D. , Chauhan I. N. , Bhatia Y. A. , Sutariya P. V. , and Rajput C. K. , A Study for Clinical Assessment of the Relationship Between Freeway Space and Various Occlusal Parameters, National Journal of Integrated Research in Medicine. (2019) 10, no. 4.

[12] Priya P. , Singh A. , and Chug A. , Restorative and Periodontal Outcomes of Deep Margin Elevation in Posterior Teeth: A Systematic Review, Cureus. (2025) 17, no. 11, e97278, 10.7759/cureus.97278. PMC12716966 41426911

[13] Samartzi T. K. , Papalexopoulos D. , Sarafianou A. , and Kourtis S. , Immediate Dentin Sealing: A Literature Review, Clinical, Cosmetic and Investigational Dentistry. (2021) 13, 233–256, 10.2147/CCIDE.S307939. PMC8232880 34188553

[14] Bresser R. , Insights Into Restoring Teeth With Deep Preparation Outline: Thinking Outside the Box, 2024, University of Groningen, 10.33612/diss.984234456.

[15] Al-Turki L. E. , Alsulimani O. A. , Baik K. M. , Othmani H. , Alqarni N. M. , Alqarni A. A. , and Al-Dabbagh R. A. , The Influence of Deep Margin Elevation and Immediate Dentin Sealing on the Fracture Strength of Premolars Restored With Indirect Inlays: An In Vitro Study, Clinical and Experimental Dental Research. (2025) 11, no. 3, e70161, 10.1002/cre2.70161, 40553010. PMC12186461 40553010

[16] Sengottaiyan A. K. , Bennani V. , and Veerasamy A. , Influence of Tooth Preparation Design on Margin Discrepancy and Internal Gap in Digitally Fabricated Fixed Complete Coverage Zirconia Prostheses: A Systematic Review of In Vitro Studies, Journal of Prosthetic Dentistry. (2025) 134, no. 3, 616–627, 10.1016/j.prosdent.2025.04.030, 40383701. 40383701

[17] Baig M. R. , Qasim S. S. , and Baskaradoss J. K. , Marginal and Internal Fit of Porcelain Laminate Veneers: A Systematic Review and Meta-Analysis, Journal of Prosthetic Dentistry. (2024) 131, no. 1, 13–24, 10.1016/j.prosdent.2022.01.009, 35260253. 35260253

[18] Palaskar J. N. , Hindocha A. D. , Jadhav P. D. , and Hasabnis P. P. , Proposing a Revised Hanau′s Formula for Determining Lateral Condylar Guidance: A Comparative Study, Journal of Oral Biology and Craniofacial Research. (2025) 15, no. 6, 1291–1296, 10.1016/j.jobcr.2025.08.014, 40895265. PMC12390920 40895265

[19] Cortes A. R. , Digital Versus Conventional Workflow in Oral Rehabilitations: Current Status, Applied Sciences. (2022) 12, no. 8, 10.3390/app12083710.

[20] Bernauer S. A. , Zitzmann N. U. , and Joda T. , The Complete Digital Workflow in Fixed Prosthodontics Updated: A Systematic Review, Healthcare. (2023) 11, no. 5, 10.3390/healthcare11050679, 36900684. PMC10001159 36900684

[21] Yang S. , Revilla-León M. , Wei C. , Yuan Q. , Yue L. , and Li J. , Accuracy (Trueness and Precision) of 3-Dimensional Virtual Patients: An In Vitro Investigation of Different Facial Scanners and Digital Integration Techniques, Journal of Dentistry. (2025) 154, 105567, 10.1016/j.jdent.2025.105567, 39805492. 39805492

[22] Lepidi L. , Kim B. C. , Giberti L. , Suriano C. , Li J. , and Grande F. , The 4D Virtual Patient: A Proof of Concept in Digital Dentistry, Journal of Prosthetic Dentistry. (2025) 134, no. 5, 1442–1445, 10.1016/j.prosdent.2024.02.029. 38616156

[23] Rasheed R. K. , Mansoor N. S. , Mohammed N. H. , and Qasim S. S. , Subtractive and Additive Technologies in Fixed Dental Restoration: A Systematic Review, Journal of Techniques. (2023) 5, no. 4, 162–167, 10.51173/jt.v5i4.1034.

[24] Tordiglione L. , De Franco M. , and Bosetti G. , The Prosthetic Workflow in the Digital Era, International Journal of Dentistry. (2016) 2016, 9823025, 10.1155/2016/9823025, 27829834. PMC5088314 27829834

